# Bacterial Lipopolysaccharide Induced Alterations of Genome-Wide DNA Methylation and Promoter Methylation of Lactation-Related Genes in Bovine Mammary Epithelial Cells

**DOI:** 10.3390/toxins11050298

**Published:** 2019-05-24

**Authors:** Jingbo Chen, Yongjiang Wu, Yawang Sun, Xianwen Dong, Zili Wang, Zhu Zhang, Yanli Xiao, Guozhong Dong

**Affiliations:** 1 College of Animal Science and Technology, Southwest University, Beibei 400716, China; c10417511@email.swu.edu.cn (J.C.); wuyongjiang@email.swu.edu.cn (Y.W.); syaw507@swu.edu.cn (Y.S.); wzl9698@swu.edu.cn (Z.W.); b20020901102@swu.edu.cn (Z.Z.); 2 Institute for Herbivorous Livestock Research, Chongqing Academy of Animal Science, Chongqing 402460, China; chenghuafen@email.swu.edu.cn; 3 College of International Studies, Southwest University, Beibei 400716, China; xyl616856826@email.swu.edu.cn

**Keywords:** lipopolysaccharide, endotoxin, DNA methylation, bovine, mammary, epithelial cells

## Abstract

Bacterial lipopolysaccharide (LPS) could result in poor lactation performance in dairy cows. High methylation of DNA is associated with gene repression. However, it is unclear whether LPS could suppress the expression of lactation-related genes by inducing DNA methylation. Therefore, the objective of this study was to investigate the impact of LPS on genome-wide DNA methylation, using methylated DNA immunoprecipitation with high-throughput sequencing (MeDIP-seq) and on the promoter methylation of lactation-related genes using MassArray analysis in bovine mammary epithelial cells. The bovine mammary epithelial cell line MAC-T cells were treated for 48 h with LPS at different doses of 0, 1, 10, 100, and 1000 endotoxin units (EU)/mL (1 EU = 0.1 ng). The results showed that the genomic methylation levels and the number of methylated genes in the genome as well as the promoter methylation levels of milk genes increased when the LPS dose was raised from 0 to 10 EU/mL, but decreased after further increasing the LPS dose. The milk gene mRNA expression levels of the 10 EU/mL LPS treatment were significantly lower than these of untreated cells. The results also showed that the number of hypermethylated genes was greater than that of hypomethylated genes in lipid and amino acid metabolic pathways following 1 and 10 EU/mL LPS treatments as compared with control. By contrast, in the immune response pathway the number of hypomethylated genes increased with increasing LPS doses. The results indicate LPS at lower doses induced hypermethylation of the genome and promoters of lactation-related genes, affecting milk gene mRNA expression. However, LPS at higher doses induced hypomethylation of genes involved in the immune response pathway probably in favor of immune responses.

## 1. Introduction 

In practical dairy production, dairy cows are often fed high-concentrate diets to meet the energy demand for high milk production or due to an intermittent forage shortage. Feeding high-concentrate diets often result in a decline in rumen pH values [1,2,3]. As a result, ruminal acidosis, especially subacute ruminal acidosis (SARA), occurs frequently in dairy cows [1,2,3,4]. Under such circumstances, endotoxin (lipopolysaccharide, LPS), a cell wall component of Gram-negative bacteria, is released in an increasing amount in the rumen [1,2,3,5]. Several studies showed when dairy cows were fed high-concentrate diets, both ruminal and circulating LPS increased [6,7,8,9,10,11]. Studies also demonstrated feeding dairy cows high-concentrate diets resulted in increased LPS concentrations in the mammary artery plasma as compared with cows fed with low-concentrate diets [12,13]. Besides high-concentrate diets, many other factors can also result in increased entry of LPS into the body and these factors include poor hygienic environments [14,15] and diseases such as mastitis and metritis [16,17].

Many studies have showed that entry of LPS into the circulation causes systemic inflammatory response. Zhou et al. also showed that when cows were fed high-concentrate diets, as compared with cows fed low-concentrate diets, local inflammatory response in the mammary gland increased, accompanying an increase in ruminal and fecal LPS [5]. When the mammary gland is exposed to LPS, it might also cause epigenetic alterations in the mammary epithelial cells, which could modify the expression of lactation-related genes, resulting in reduced milk gene expression in the mammary epithelial cells. 

Epigenetics is the study of heritable changes in gene expression that are independent of DNA sequences [18]. Major genetic evens include histone modification, DNA methylation, and microRNA regulation. DNA methylation is the oldest epigenetic mechanism that is known to associate with gene repression [19]. DNA methyltransferases (DAMTs) catalyze DNA methylation, in which a methyl group from *S*-adenosyl methionine (*S*AM) is transferred to the carbon 5′ position of cytosine on the DNA template [20,21]. DNA methylation occurs predominantly in cytosine-phosphate-guanine (CpG) dinucleotides in mammals [22]. The methylation level of DNA in promoter regions of specific genes efficiently regulates the transcription of the corresponding genes. High methylation level tends to decrease the access of specific transcriptional factors to the promoter region of genes, whereas low or absent methylation leads to an increased accessibility and transcriptional activity [23,24]. 

DNA methylation occurs in response to environmental factors, including diet, toxins, pollutants, heat stress, disease and so forth. Bacterial endotoxin might have profound impacts on gene expression in the mammary epithelial cells through DNA methylation modifications. We thus hypothesized that LPS may induce DNA methylation, resulting in reduced milk gene expression in the bovine mammary epithelial cells. Therefore, the objective of this study was to explore the effects of LPS on the genome-wide DNA methylation and promoter methylation of lactation-related genes in bovine mammary epithelial cells, so as to provide an insight into the epigenetic mechanisms of the adverse effects of LPS on milk gene expression of the bovine mammary epithelial cells. In the present study, the doses of LPS used to culture with the bovine mammary epithelial cells were set to cover a wide range of plasma LPS concentrations in cows under different conditions that vary from healthy status through SARA and diseases. While LPS might not be detected in the plasma of healthy cows, it was present in the plasma with levels ranging from 0.2 to 860 endotoxin units (EU)/mL (1 EU = 0.1 ng) in cows fed high-concentrate diets [6,7,8,9,10,11,12,13] or in mastitis and metritis cows [16,17]. 

## 2. Results

### 2.1. Effect of LPS on Genome-Wide DNA Methylation in Bovine Mammary Epithelial Cells

**Figure 1:**
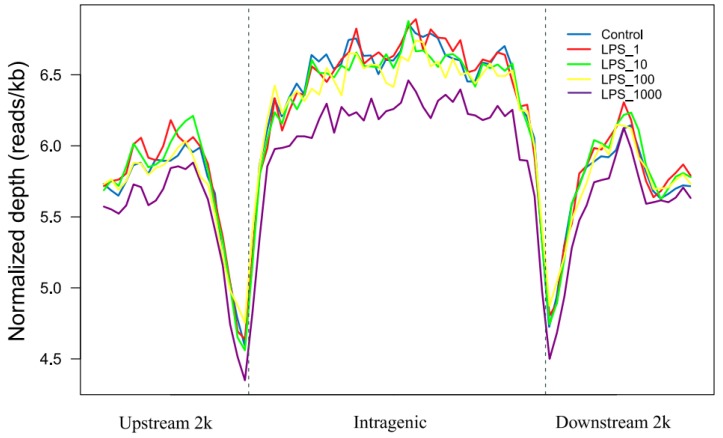
Distribution of MeDIP-seq reads in the intragenic region and the regions 2 kb upstream and downstream in MAC-T bovine mammary epithelial cells under different treatments of lipopolysaccharide (LPS). The *x*-axis indicates the position of reads in different genomic regions, and the *y*-axis indicates the normalized read number. The treatments were as follows: Control, without LPS; LPS_1, 1 EU/mL; LPS_10, 10 EU/mL; LPS_100, 100 EU/mL; and LPS_1000, 1000 EU/mL.

In this study, methylated DNA immunoprecipitation with high-throughput sequencing (MeDIP-seq) was used to determine the genomic DNA methylation in MAC-T bovine mammary epithelial cells. Distribution of MeDIP-seq reads representing the methylation level in different genomic regions is shown in Figure 1. The reads were distributed in components of the genome, which include the upstream 2 kb region, 5′-untranslated regions (UTRs), coding DNA sequences (CDSs), introns, 3′-UTRs, and the downstream 2 kb region. The region from the transcript start site (TSS) to the transcript termination site (TTS) is defined as the intragenic region. The results showed the total reads in the intragenic region were greater than the results in the regions upstream 2 kb and downstream 2 kb among all treatments. Two valleys of DNA methylation were in the upstream region adjacent to the TSS and the downstream region adjacent to the TTS, respectively. 

**Figure 2:**
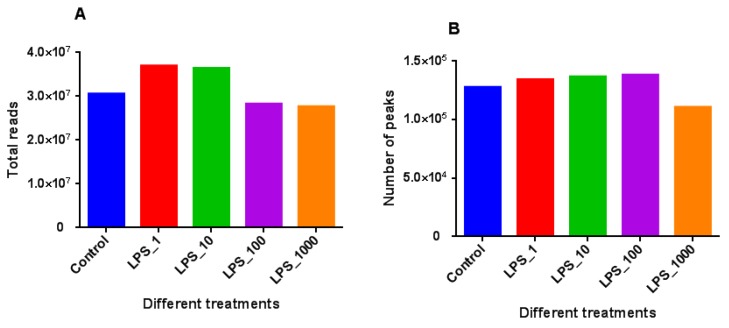
DNA methylation profiles in MAC-T bovine mammary epithelial cells under different lipopolysaccharide (LPS) treatments. Changes in genomic methylation levels were expressed as total reads (**A**) and the number of methylated peaks (**B**). The treatments were as follows: Control, without LPS; LPS_1, 1 EU/mL; LPS_10, 10 EU/mL; LPS_100, 100 EU/mL; and LPS_1000, 1000 EU/mL.

The number of reads was higher in treatments with 1 or 10 EU/mL LPS, as compared with control (Figure 2). The number of reads was lower when the dose of LPS increased to 100 or 1000 EU/mL. The global DNA methylated peaks were higher in treatments with 1, 10 or 100 EU/mL LPS, but lower in the treatment of 1000 EU/mL LPS.

**Figure 3:**
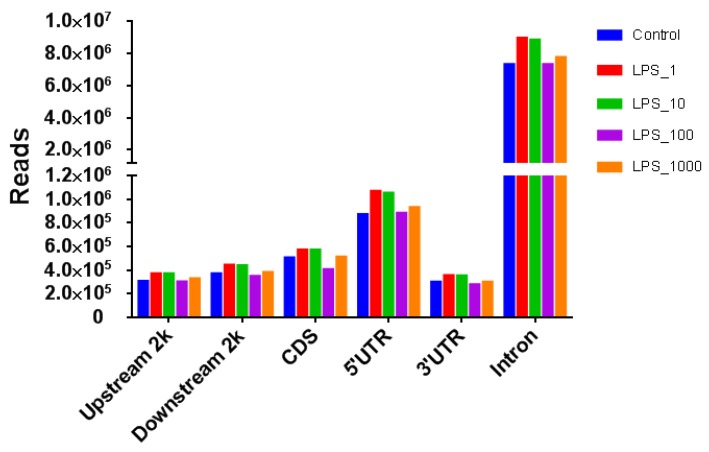
Distribution of MeDIP-seq reads in different genomic components of MAC-T bovine mammary epithelial cells under different lipopolysaccharide (LPS) treatments. The treatments were as follows: Control, without LPS; LPS_1, 1 EU/mL; LPS_10, 10 EU/mL; LPS_100, 100 EU/mL; and LPS_1000, 1000 EU/mL. Upstream 2k: the 2 kb upstream region; Downstream2k: the 2 kb downstream region; CDS: coding DNA sequence; 5′UTR: 5′-untranslated region; and 3′UTR: 3′-untranslated region.

**Figure 4:**
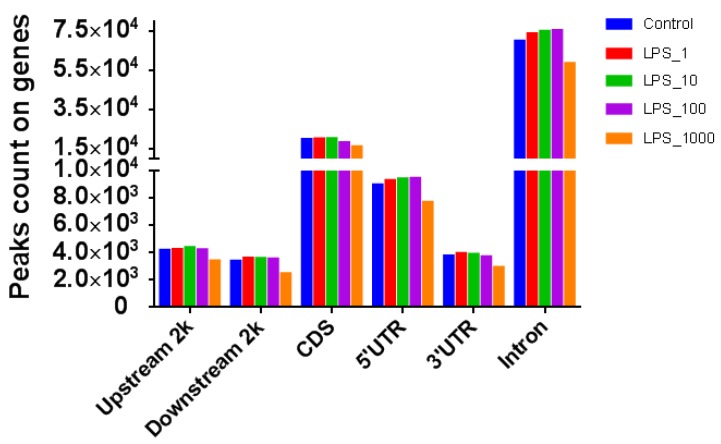
Distribution of methylated gene numbers in different genomic components of MAC-T bovine mammary epithelial cells under different lipopolysaccharide (LPS) treatments. The treatments were as follows: Control, without LPS; LPS_1, 1 EU/mL; LPS_10, 10 EU/mL; LPS_100, 100 EU/mL; and LPS_1000, 1000 EU/mL. Upstream 2k: the 2 kb upstream region; Downstream2k: the 2 kb downstream region; CDS: coding DNA sequence; 5′UTR: 5′-untranslated region; and 3′UTR: 3′-untranslated region.

The number of reads in different genomic components with the treatment of 1 or 10 EU/mL LPS was higher than that of the control, whereas the treatments with 100 or 1000 EU/mL LPS did not increase the number of reads compared with control (Figure 3). The number of methylated genes in all the components except the CDSs increased with almost all LPS treatments except the 1000 EU/mL LPS treatment (Figure 4). 

**Figure 5:**
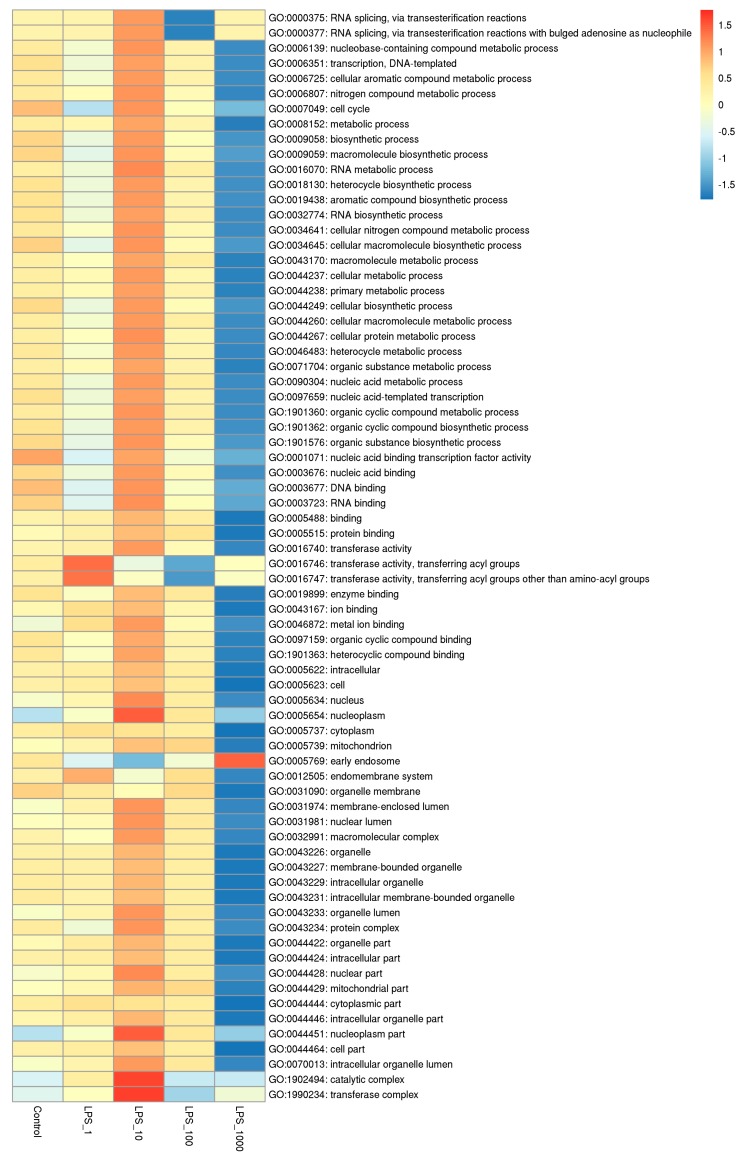
The numbers of methylated genes in the upstream 2k region in gene ontology (GO) function analysis under different lipopolysaccharide (LPS) treatments. Each column represents a treatment and each row represents a GO pathway. The number of methylated genes in the pathway is expressed in red-blue color gradients from high to low. The treatments were as follows: Control, without LPS; LPS_1, 1 EU/mL; LPS_10, 10 EU/mL; LPS_100, 100 EU/mL; and LPS_1000, 1000 EU/mL.

**Figure 6:**
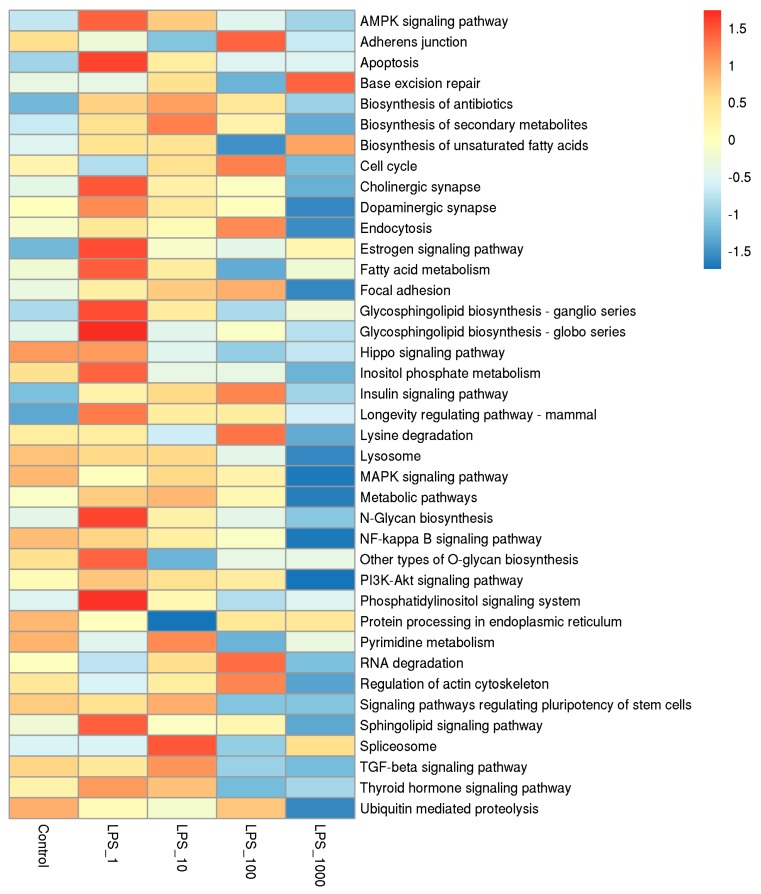
Numbers of methylated genes in the 2 kb upstream region in Kyoto Encyclopedia of Genes and Genomes (KEGG) metabolic pathway analyses under different lipopolysaccharide (LPS) treatments. Each column represents a treatment and each row represents a GO pathway. The number of methylated genes in the pathway is expressed in red-blue color gradients from high to low. The treatments were as follows: Control, without LPS; LPS_1, 1 EU/mL; LPS_10, 10 EU/mL; LPS_100, 100 EU/mL; and LPS_1000, 1000 EU/mL.

CpG islands are mainly densely distributed in the promoter which is principally located in the 2 kb upstream region. We analyzed gene ontology (GO) function and Kyoto Encyclopedia of Genes and Genomes (KEGG) pathway enrichment of peak related genes in the upstream 2k region of the TSS. The pathways of adjusted *p* < 0.05 were shown in heat maps. In the GO functional enrichment pathways, the number of methylated genes obviously increased when cells were treated with 10 EU/mL LPS, whereas the number of methylated genes was lowest when treated at 1000 EU/mL of LPS (Figure 5; Table S1). In the KEGG pathways, the number of methylated genes was the highest when cells were treated with 1 EU/mL LPS, whereas that of methylated genes was the lowest with the 1000 EU/mL LPS treatment (Figure 6; Table S2). 

The numbers of hypermethylated and hypomethylated genes in the 2 kb upstream region of selected pathways are presented in Table 1. The hypermethylated genes were more than the hypomethylated genes in lipid and amino acid metabolic pathways, following 1 and 10 LPS treatments as compared with the control, while the hypermethylated genes were fewer than hypomethylated genes in lipid and amino acid metabolic pathways following 100 and 1000 LPS treatments in comparison with control. In the immune response pathway, the number of hypermethylated genes among LPS treatments was almost the same, whereas that of hypomethylated genes increased with increasing LPS doses. 

### 2.2. Effects of LPS on Promoter Methylation of Lactation-Related Genes in Bovine Mammary Epithelial Cells

Since the promoter is more directly involved in the control of gene transcription, based on the genomic DNA methylation information we are also interested in exploring the effects of LPS on promoter methylation of specific milk genes. Primers were designed for 9 major genes (*FASN*, *SCD*, *STAT5A*, *S6K1*, *ACSL1*, *ACACA*, *ACSS1*, *ACSS2*, and *FADS2*) involved in milk fat and protein syntheses and MassArray was used to determine the promoter methylation levels of these genes. Four genes (*FASN*, *ACACA*, *ACSS2*, and *S6K1*) were successfully amplified using PCR and the other five could not be amplified. The unsuccessful amplification could be due to failures in designing high quality primers since these genes probably do not possess sites in the CpG islands for suitable primer designs. 

**Figure 7:**
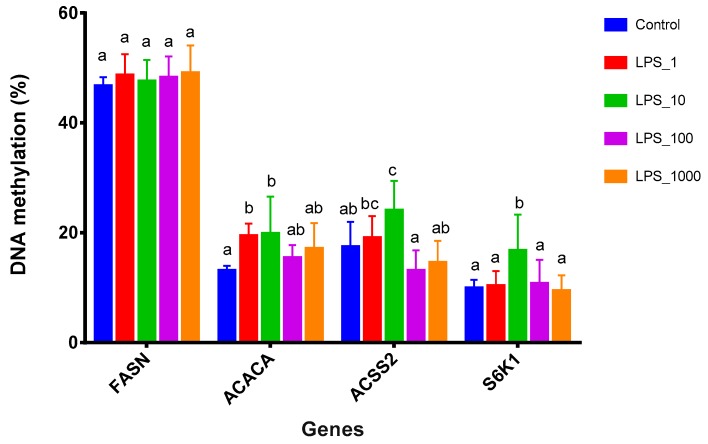
Effects of different LPS treatments on methylation of CpG islands in the promoter region of lactation-related genes in MAC-T bovine mammary epithelial cells. The treatments were as follows: Control, without LPS; LPS_1, 1 EU/mL; LPS_10, 10 EU/mL; LPS_100, 100 EU/mL; and LPS_1000, 1000 EU/mL. Data represent the mean and standard deviation (n = 6/treatment). Columns with different lowercase letters (a,b,c) indicate significantly different values among treatments (*p* < 0.05). *FASN*: fatty acid synthase; *ACACA*: acetyl-CoA carboxylase 1; *ACSS2*: acyl-CoA synthetase short-chain family member 2; *S6K1*: ribosomal protein S6 kinase 1.

The MassArray results showed that the methylation levels of *FASN* were not significantly (*p* > 0.05) different among all the treatments (Figure 7). The methylation levels of *ACACA*, *ACSS2*, and *S6K1* increased (*p* < 0.05) after LPS doses were raised from 0 through 1 or 10 EU/mL. When the LPS doses were further increased to 100 and 1000 EU/mL, the methylation levels of these genes did not increase (*p* > 0.05), as compared with the control. 

### 2.3. Effects of DNA Methylation on Gene Expression

**Figure 8:**
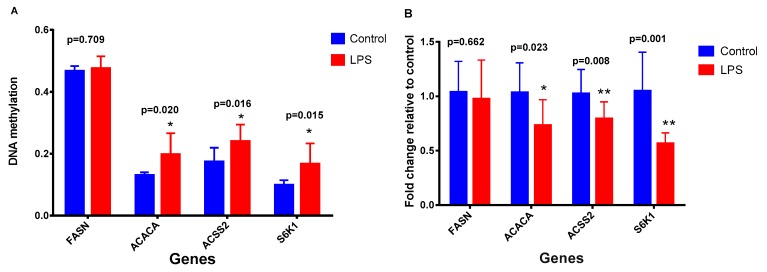
DNA methylation (**A**) and mRNA expression (**B**) of lactation-related genes in MAC-T bovine mammary epithelial cells. The cells were treated either without lipopolysaccharide (LPS) or with LPS at 10 EU/mL. Data represent the mean and standard deviation (n = 4/treatment) and the asterisk indicates statistical difference between the indicated column and the control column (*, *p* < 0.05; **, *p* < 0.01). *FASN*: fatty acid synthase; *ACACA*: acetyl-CoA carboxylase 1; *ACSS2*: acyl-CoA synthetase short-chain family member 2; *S6K1*: ribosomal protein S6 kinase 1.

To verify the correlation between DNA methylation and gene expression, we determined the promoter methylation levels of *FASN*, *ACACA*, *ACSS2*, and *S6K1*. In the meantime, their mRNA expression was also measured (Figure 8). Since the dose of 10 EU/mL LPS generally had the highest methylation levels among the lactation-related genes (Figure 7), we then compared the methylation levels of *FASN*, *ACACA*, *ACSS2*, and *S6K1* with their mRNA expression levels after cells were treated with 10 EU/mL LPS. The results revealed that the gene methylation levels were negatively correlated with mRNA expression levels, i.e., gene hypermethylation was associated with down-regulated mRNA expression.

## 3. Discussion

DNA methylation is one of the central epigenetic controls of gene transcription. In the present study, we quantified the DNA methylation levels in different genomic regions after incubating the MAC-T bovine mammary epithelial cells with LPS. We found that the total MeDIP-seq reads of the intragenic region were higher that the regions upstream and downstream regardless of the treatments. These genomic DNA methylation distribution patterns are consistent with the findings of other studies using blood lymphocytes of cows [25] and placental tissues of cattle [26] as well as the heart tissue of chickens [27]. The study by Su et al. is the first to map bovine DNA methylome, in which the methylation levels in the gene body were relatively high, whereas the upstream region remained hypomethylated [26]. Under normal conditions, although the total MeDIP-seq reads in the upstream region is low, however, the upstream region is more easily subjected to changes in the methylation status. Therefore, the methylation level of the upstream region is most closely associated with gene expression regulation [26]. In our study, we further found that the genomic methylation in different regions including the upstream 2 kb region increased when the LPS doses were raised from 0 to 1 or 10 EU/mL, but these did not increase, or even decreased after further increasing the LPS dose. Our results suggest that the impact of LPS on genomic DNA methylation is dose dependent.

Our results further showed that the number of hypermethylated genes was greater than hypomethylated genes in lipid and amino acid metabolic pathways following 1 or 10 EU/mL LPS treatments as compared with control. In the immune response pathway, the number of hypermethylated genes among LPS treatments was almost the same, whereas that of hypomethylated genes increased with increasing LPS doses. The results suggest that LPS could suppress milk gene expression at lower doses, but increase expression of immune genes at higher doses. In support of this idea, it was reported that feeding high-concentrate diets resulted in a level of about 1 EU/mL in the mammary artery plasma and an increase in methylation levels of genes (*ACSL1*, *FASN* and *SCD*) associated with milk fat synthesis in the mammary tissue of dairy cows [13]. In addition, another study demonstrated that high-grain feeding led to a level of 0.86 EU/mL in the mammary artery plasma, which was associated with a decrease in gene expression of αs1-casein (*CSN1S1*), β-casein (*CSN2*), the mammalian target of rapamycin (*mTOR*), and ribosomal protein S6 kinase (*S6K*) in the mammary gland of lactating dairy cows [12]. With respect to the immune response, it was reported that when LPS doses were below 10 EU/mL, the mRNA expression of cytokines genes (*IL-1β*, *IL-6* and *IL-8*) was not different from that of control in the mammary tissue of dairy cows, whereas the mRNA expression of these inflammatory genes was significantly higher when the LPS dose was further increased to 100 and 1000 EU/mL [28]. 

The result of our present study has implications for practical dairy production. Dairy cows are commonly fed high-concentrate diets in practical dairy production. Under the condition, LPS is usually present in the plasma at a level of about 1 EU/mL [6,7,8,10,11,12,13]. Therefore, high-concentrate feeding may not facilitate a high milk gene expression in mammary gland. In fact, the milk fat and protein contents as well as milk fat and protein yields in cows fed high-concentrate diets decreased [5,13,29]. 

Since the promoter is more directly involved in the control of gene transcription, we further determined the promoter methylation levels of specific milk genes involved in milk fat and protein synthesis. In agreement with the genomic DNA methylation results, the promoter methylation levels of lactation-related genes, such as *ACACA*, *ACSS2* and *S6K1*, increased when the LPS dose were raised from 0 to 1 or 10 EU/mL, but decreased after further increasing the LPS dose. We further compared the promoter methylation levels of these genes with their mRNA expression levels measured at the LPS dose of 10 EU/mL, and the results revealed that the gene methylation levels were negatively correlated with mRNA expression levels, i.e., gene promoter hypermethylation was associated with down-regulated mRNA expression. Similarly, a study showed that lower DNA methylation level, measured as a percentage of 5′ methyldeoxycytidine in the mammary tissue of Holstein cows, was associated with an increase in *CSN2* mRNA [30]. In another study, Nguyen et al. reported that the methylation level in the upstream region of the *CSN1S1* gene in the mammary gland was negatively correlated with *CSN1S1* and *CSN2* mRNA as well as milk protein secretion [31]. Vanselow et al. also showed that DNA-remethylation around a signal transducer and activator of transcription 5 (STAT5)-binding enhancer in the *CSN1S1* promoter was associated with abrupt shutdown of the CSN1S1 protein synthesis during acute mastitis [32]. 

It is worth mentioning that the promoter methylation levels of lactation-related genes decreased at higher LPS doses such as 100 and 1000 EU/mL, which might increase theoretically gene expression of milk genes. However, this may not translate into actual higher milk fat and protein secretion in the mammary gland, since higher inflammatory responses were mounted at higher LPS doses, and as a matter of fact, the milk fat and protein secretion was reduced in the mammary tissue treated with 100 or/and 1000 EU/mL LPS, as compared with control and lower LPS treatments [28]. The reason why the promoter methylation levels of lactation-related genes decreased at higher LPS doses remains unclear and warrants further investigation. 

## 4. Conclusions

In the present study involving bovine mammary epithelial cells, we demonstrated the genomic methylation and promoter methylation of lactation-related genes in bovine epithelial cells increased when the LPS dose were raised from 0 to 1 or 10 EU/mL, but decreased after further increasing the LPS dose. The number of hypermethylated genes was more than that of hypomethylated genes in lipid and amino acid metabolic pathways following 1 and 10 EU/mL LPS treatments as compared with untreated cells. While in the immune response pathway the number of hypermethylated genes was not affected by LPS treatments, the number of hypomethylated genes increased with increasing LPS doses. The results suggest that LPS at lower doses induced hypermethylation of the genome and the promoters of lactation-related genes, affecting milk gene mRNA expression. However, LPS at higher doses induced hypomethylation of genes involved in the immune response pathway probably in favor of immune responses. 

## 5. Materials and Methods

### 5.1. Research Ethics

This study did not involve the use of animals. The bovine mammary epithelial cell line (MAC-T cells) used in this experiment was provided by Professors Jianxin Liu and Hongyun Liu at the Institute of Dairy Science, Zhejiang University, China. The cell line establishment methods were as described by Huynh et al. [33]. 

### 5.2. Cell Culture and Treatments 

The composition of the complete culture medium is as follows: fetal bovine serum (10 mL; Gibco, 10099-141, Grand Island, NY, USA), epidermal growth factor (0.5 μg; Sigma, E4127, Saint Louis, MO, USA), insulin-transferrin-selenium (250 μg; Gibco, 51500-056, Grand Island, NY, USA), hydrocortisone (0.1 mg; Solarbio, G8450, Beijing, China), progesterone (100 μg; Solarbio, p9060, Beijing, China), and DMEM/F12 medium (90 mL; Gibco, 11330-032, Grand Island, NY, USA). MAC-T cells were seeded at a density of 2 × 10^5^ per mL in 6-well cell culture plates, 2 mL medium was added to each well of the 6-well cell culture plate, and the medium was replaced every 24 h. The cell culture was maintained at 37 °C and 5% CO_2_ in a CO_2_ incubator (Thermo Fisher Scientific, M371, Waltham, MA, USA). The MAC-T cells used in this study were checked for their viability to ensure the growth curve of the cells was normal and satisfactory (Figure S1). 

In the first experiment, the MAC-T bovine epithelial cells were incubated (n = 6) with LPS (*Escherichia coli* O111:B4; Sigma, L2630, Saint Louis, MO, USA) at doses of 0, 1, 10, 100, 1000 EU/mL, respectively. The doses of LPS were set to cover a wide range (0–860 EU/mL) of plasma LPS concentrations in cows under different conditions that vary from healthy status through SARA and diseases [6,7,8,9,10,11,12,13,16,17]. DNA was extracted after 48 h culture and used for genome-wide methylation assay.

In the second experiment, MAC-T cells were cultured (n = 6) with five doses of LPS (0, 1, 10, 100, and 1000 EU/mL, respectively), as described above in the first experiment. DNA was extracted after 48 h culture and the promoter methylation levels of specific milk genes was measured. 

In the third experiment, the treatments of MAC-T cells (n = 4) were as follows: control and LPS (10 EU/mL). The results of the present study, as described above in Figure 7, showed LPS at the dose of 10 EU/mL increased DNA methylation levels most effectively. RNA was extracted after 48 h culture and the milk gene mRNA expression levels were determined using real-time qPCR.

### 5.3. DNA Extraction and Evaluation 

After 48 h treatment, the cells were washed with DPBS (Solarbio, D1040-500, Bejing, China) twice, digested with 0.25% trypsin/EDTA (Gibco, 25200056, Grand Island, NY, USA), and centrifuged to obtain clean cells. For genome-wide methylation measurements, 6 samples per treatment were pooled for DNA extraction. For gene promoter methylation measurements, individual sample per treatment was used to extract DNA. 

Total DNA was extracted using a global genomic DNA kit (DP304, Tiangen, Bejing, China) by following the manufacturer’s protocols, and was quantified using micro-spectrophotometer (NanoDrop2000, Thermo Fisher Scientific). The concentration of DNA was assayed by a fluorometer (Qubit, Thermo Fisher Scientific) and the DNA integrity was measured by agarose gelelectrophoresis (Figure S2). 

### 5.4. Assay for Genome-Wide Methylation 

Genome-wide methylation was measured by MeDIP-seq assay. Genomic DNA was sonicated to generate 100–500 bp fragments. DNA repair, addition of bases at 3′-terminal and adaptor ligation were performed, using Paired-End DNA Sample Prep Kit (Illumina Inc., San Diego, CA, USA) by following the manufacturer’s protocols. The double-stranded DNA was denatured. 5′-mC antibodies were used to acquire methylated DNA fragment enrichment, which was verified by qPCR. After PCR amplification, gel electrophoresis was performed and the band was excised. Bands of 220–320 bp were recovered and scanned using DNA1000 chips. Qualified libraries are then used for sequencing. The sequencing results were compared with reference genomes and the sequences with the sole position were subjected to subsequent analyses. Model-based analysis of ChIP-Seq (MACS1.4.0) was used to scan the enriched peaks where the reads were mapped to the same position in the genome, and the analysis included the genomic distribution trend of MeDIP-seq data, calculation of MeDIP-seq enrichment regions (peaks), and difference analysis between sample-based peaks. According to the MeDIP-seq assay, only the sonicated fragments with DNA methylation occurring at a certain region can be precipitated. Therefore, the fragments detected by high-throughput sequencing represented those that were methylated. 

### 5.5. Assay for Promoter Methylation of Milk Genes 

The putative CpG islands of the milk genes were analyzed in an upstream 5000 bp to the downstream 1000 bp sequence of the TSS using CpG islands searcher (http://www.ebi.ac.uk/Tools/seqstats/emboss_cpgplot/). The primer was designed by Agena EpiDesigner, and the primers of CpG islands in the promoter of lactation-related genes were presented in Table 2. There are no CpG islands in the promoter of *CSN1S1* due to a lack of CpG dense regions, therefore no primers were designed. The DNA samples were treated with bisulfite using the EZ DNA Methylation Gold kit (ZYMO RESEARCH CORP., Irvine, CA, USA) by following the manufacturer’s instructions. PCR amplification of the CpG islands region was conducted under the following conditions: 94 °C, 4 min; 35 cycles of 94 °C, 20 sec; 55 °C,30 sec, 72 °C, 1 min. PCR production was then performed with Agena MassArray system (Agena Bioscience, San Diego, CA, USA) according to manufacturer’s guidelines. Percentage of methylation of each CpG unit or single CpG site per product was calculated using MassArray EpiTYPER software.

### 5.6. RNA Isolation and cDNA Synthesis

Total RNA was isolated by using Trizol (Thermo Fisher Scientific, 15596026, Waltham, MA, USA). The adherent cells were washed three times with DPBS (Solarbio, D1040-500, Beijing, China), then 1 mL of Trizol was added into each well to lyse cells for 5 min, and the cell suspension was transferred into a 1.5 mL centrifugal tube. 0.2 mL chloroform was added to each sample, shaking and mixing for 30 seconds. The mixture was maintained still for 3 min and then centrifuged for 15 min at 12,000 *g* and 4 °C. After centrifugation, the liquid was divided into three layers, and the top layer was carefully transferred to a centrifugal tube. The same amount of isopropanol solution (about 0.4 mL) was added into it, and the mixture was kept still at room temperature for 10 min, and then centrifuged at 12,000 *g* and 4 °C for 10 min. After abandoning the upper solution, 1 mL 75% ethanol solution was added. It was then shaken thoroughly and centrifuged at 7500 *g* and 4 °C for 5 min. After discarding the upper solution, the white precipitation at the bottom of the centrifugal tube, equivalent to the isolated RNA, was harvested. The cap of the centrifugal tube was opened to make ethanol completely volatilize to avoid interference in the subsequent amplification experiments, and finally the RNA was dissolved with RNase/DNase free water, and its purity and concentration were determined. The RNA was used for the preparation of cDNA using the iScript cDNA synthesis kit (Bio-Rad, 1708891, Hercules, CA, USA) by following manufacturer’s protocols.

### 5.7. Quantitative Real-Time PCR

The prepared cDNA was amplified with Ssofast EvaGreen Supermix (Bio-Rad, 1725201, Hercules, CA, USA) and gene-specific primers in final reaction mixture of 20 μL. Gene-specific primer pairs were designed using Primer Premier 5.0 software (Table 3). The glyceraldehyde-3-phosphate dehydrogenase gene (*GAPDH*) was used as the internal control gene. Amplification and quantification were performed with the C1000 Thermal Cycler (BIO-RAD CFX96 Real-Time System, Hercules, CA, USA) under the following cycling conditions: pre-incubation at 95 °C for 30 sec, followed by 40 cycles of denaturation at 95 °C for 5 sec and annealing at 58 °C for 5 sec. Melting curves for each gene were analyzed to ensure the amplification and generation of single PCR products. Calculations of relative expression levels were performed using the 2^−ΔΔCT^ method [34]. 

### 5.8. Statistical Analyses

For the analysis of MeDIP-seq data, methylated peaks of each genomic region for each differentiated methylated gene were merged and the number of reads for each sample was subjected to difference analysis using a chi-square test, and p values were corrected by FDR test to avoid false positives. According to differences in reads, each differentially methylated region was divided into up and down regions identified using a filter standard of *p* < 0.01. The orthologous differentially methylated genes were submitted to the Database for Annotation, Visualization, and Integrated Discovery (DAVID) web server (http://david.abcc.ncifcrf.gov/) to perform functional enrichment analysis with GO and KEGG pathway categories. The differentially methylated and expressed genes were classified into cellular components, molecular functions and biological processes using GO or KEGG annotation, and the significantly different enrichment pathways were identified based on Q ≤ 0.05. 

For the MassArray and qPCR data, statistical analyses were performed to compare the mean differences in promoter methylation levels and mRNA expression levels of lactation-related genes among treatments by using One-way ANOVA of SPSS (v.22). Mean differences for all variables were separated and compared using Duncan’s multiple comparison procedure. And data were presented as means ± standard deviation. Significance was declared at *p* < 0.05, and a tendency was considered if 0.05 < *p* < 0.10.

## Figures and Tables

**Table 1:** Numbers of hypermethylated (up) and hypomethylated (down) genes in selected pathways in MAC-T bovine mammary epithelial cells.

Pathway	Methylation	Control vs. LPS_1	Control vs. LPS_10	Control vs. LPS_100	Control vs. LPS_1000
Lipid metabolism	up	30	22	13	24
	down	16	16	20	28
Amino acid metabolism	up	20	18	10	10
	down	9	12	20	27
Carbohydrate metabolism	up	17	20	23	17
	down	14	11	18	22
Immune response	up	44	41	42	44
	down	34	34	46	62
Cell growth	up	18	23	20	15
	down	18	16	22	20

The treatments were as follows: Control, without lipopolysaccharide (LPS); LPS_1, 1 EU/mL; LPS_10, 10 EU/mL; LPS_100, 100 EU/mL; and LPS_1000, 1000 EU/mL.

**Table 2:** Methylation primers for lactation-related genes.

Gene	Primer	Product Size (bp)
*FASN*	F: GTATTTGGGTTATTTTGGGGGTTAT R: CCAAAAAAACTCCTTTATTCAAACC	482
*SCD*	F: TGAATTTTTTGAAGGTAAGGATTATG R: AAACACCTAACAATTAAAATTCCCC	560
*STAT5A*	F: TTTTTTGATAGATGAGAAAATTGAGG R: ACAACAACAACAACAACAACAAAAT	532
*S6K1*	F: GAGGATTTGGTTTTTAGGTGTGAG R: AAAAAATACAACAAAACCCCCTTAC	451
*ACSL1*	F: AGGAAAGTTTTTAGGATTTTAGGGA R: CTAAAAAAAACACTCACCTCCTCC	568
*ACACA*	F: TTTTGTAGAGAGGTTTGAGAGGTTG R: TAACATCAAAATAAACCACCACATC	478
*ACSS1*	F: GATTAGTTGGTTTTTGGGAGGTTAG R: AAAAATCCCACTACTACTTCCTTCC	529
*ACSS2*	F: AATTTTGGTTAATGGGTTTTTTTGT R: ATAAACCCCCACTTCTCTCCTAACTA	453
*FADS2*	F: TTTGTTGATTATTGTGGAAATTTAGG R: AAAAAACCCCAAACCCTTACC	595

*FASN*: fatty acid synthase; *SCD*: Stearoyl-CoA desaturase; *STAT5A*: signal transducer and activator of transcription 5A; *S6K1*: ribosomal protein S6 kinase 1; *ACSL1*: acyl-CoA synthetase long-chain family member 1; *ACACA*: acetyl-CoA carboxylase 1; *ACSS1*: acyl-CoA synthetase short-chain family member 1; *ACSS2*: acyl-CoA synthetase short-chain family member 2; *FADS2*: Fatty acid desaturase 2.

**Table 3:** Fluorescence quantitative PCR gene primers.

Gene	Primer	Product Size (bp)	Accession Number
*ACACA*	F: GATCCAGGCCATGCTAAG R: CTGTTTCTCCAGCCACTC	103	XM_024979607.1
*ACSS2*	F: GGGCGAATGCCTCTACTGC R: GCTGGGTGATGATGGATGG	254	NM_001105339.1
*S6K1*	F: GGACATGGCAGGGGTGTTT R: GGTATTTGCTCCTGTTACTTTTCG	283	NM_205816.1
*FASN*	F: AGGACCTCGTGAAGGCTGTGA R: CCAAGGTCTGAAAGCGAGCTG	85	XM_005220997.3
*GAPDH*	F: GGGTCATCATCTCTGCACCT R: GGTCATAAGTCCCTCCACGA	177	NM_001034034.2

*ACACA*: acetyl-CoA carboxylase 1; *ACSS2*: acyl-CoA synthetase short-chain family member 2; *S6K1*: ribosomal protein S6 kinase 1; *FASN*: fatty acid synthase; *GAPDH*: glyceraldehyde-3-phosphate dehydrogenase.

## Supplementary Materials

The following are available online at https://www.mdpi.com/2072-6651/11/5/298/s1, Table S1: Numbers of methylated genes in the upstream 2k region in gene ontology (GO) function analysis under different lipopolysaccharide (LPS) treatments, Table S2: Numbers of methylated genes in the 2 kb upstream region in Kyoto Encyclopedia of Genes and Genomes (KEGG) metabolic pathway analyses under different lipopolysaccharide (LPS) treatments, Figure S1: Growth curve of the MAC-T bovine mammary epithelial cells, Figure S2: DNA sample integrity examined by electrophoresis.
