# Supplementary material for: Bacterial Lipopolysaccharide Induced Alterations of Genome-Wide DNA Methylation and Promoter Methylation of Lactation-Related Genes in Bovine Mammary Epithelial Cells
Source: Toxins (Basel). 2019 May 24;11(5):298. doi: 10.3390/toxins11050298 (PMC6563294; doi:10.3390/toxins11050298)
Supplement: Supplementary file 1 [file toxins-11-00298-s001.zip › toxins-485250-SI/Figure S1.pdf]

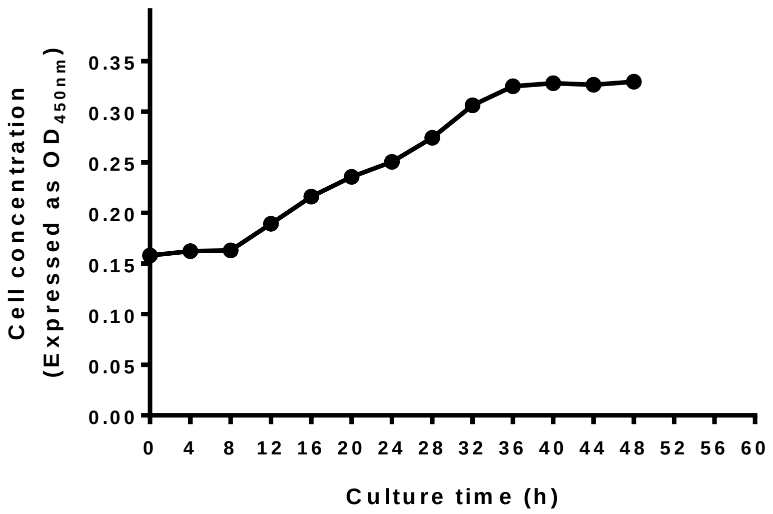

Figure S1. Growth curve of the MAC-T bovine mammary epithelial cells. The growth of cells was measured (n = 5) by using CCK-8 (Cell Counting Kit 8, Dojindo, Japan). The optical density (OD) was determined at 450 nm on a microplate reader (Bio-Rad, xMark™, USA).
