# Supplementary material for: Bacterial Lipopolysaccharide Induced Alterations of Genome-Wide DNA Methylation and Promoter Methylation of Lactation-Related Genes in Bovine Mammary Epithelial Cells
Source: Toxins (Basel). 2019 May 24;11(5):298. doi: 10.3390/toxins11050298 (PMC6563294; doi:10.3390/toxins11050298)
Supplement: Supplementary file 1 [file toxins-11-00298-s001.zip › toxins-485250-SI/Figure S2.pdf]

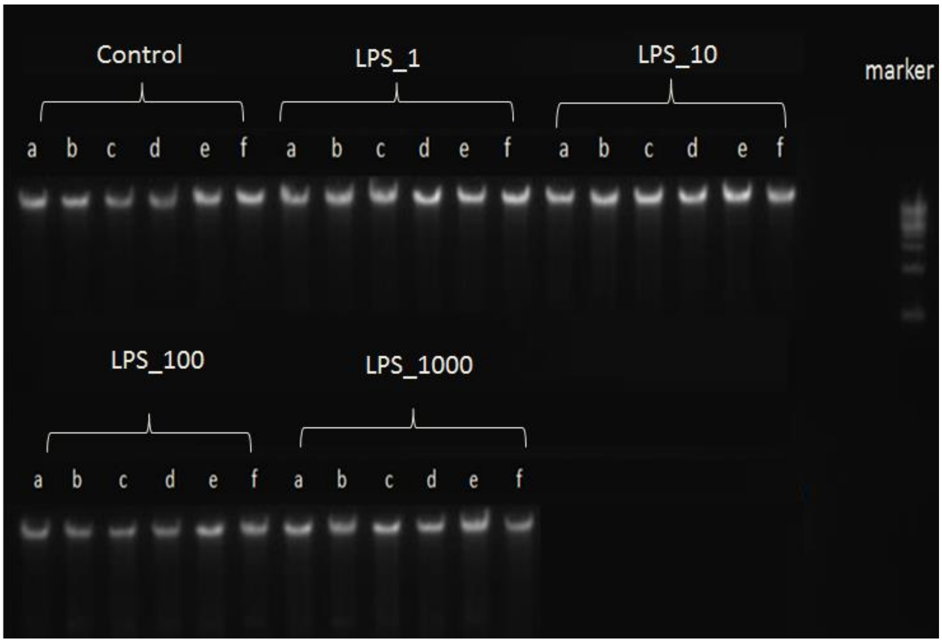

Figure S2. DNA sample integrity examined by electrophoresis. Each band represents one DNA sample and there was no degradation since no smears were seen. Control, without LPS; LPS_1, 1 EU/mL; LPS_10, 10 EU/mL; LPS_100, 100 EU/mL; and LPS_1000, 1000 EU/mL.
